# Dietary consumption of cruciferous vegetables and bladder cancer risk: A systematic review and meta-analysis

**DOI:** 10.3389/fnut.2022.944451

**Published:** 2022-08-18

**Authors:** Pengkui Yu, Lin Yu, Yi Lu

**Affiliations:** Department of Urology, Shengzhou People’s Hospital, Shengzhou Branch of the First Affiliated Hospital of Zhejiang University, Shengzhou, China

**Keywords:** cruciferous vegetables, bladder cancer, meta-analysis, cohort, risk

## Abstract

**Objective:**

Previous studies on the association of cruciferous vegetables intake with bladder cancer risk have reported inconsistent results. We performed the present meta-analysis to summarize evidence on this association and to quantify the potential dose-response relation based on all available cohort studies.

**Methods:**

A comprehensive literature search of relevant articles up to March 2022 was performed in PubMed and EMBASE. The summary risk estimates with 95% confidence intervals for the highest vs. the lowest intake of cruciferous vegetables were calculated. Dose-response meta-analysis was also performed for studies reporting categorical risk estimates for at least three quantitative levels of cruciferous vegetables intake.

**Results:**

We found that the highest cruciferous vegetables intake was not significantly associated with a lower risk of bladder cancer, compared with the lowest cruciferous vegetables intake category (RR = 0.92, 95% CI 0.80–1.06). Linear dose-response meta-analysis indicated that the pooled RRs for 10 g/day or 1 servings/week increment of cruciferous vegetables intake was not significantly associated with a reduced risk of bladder cancer (*P* = 0.106 and *P* = 0.147, respectively). There was no evidence of significant publication bias either with Begg’s test (*P* = 0.386) or Egger’s test (*P* = 0.253).

**Conclusion:**

The results of this study did not support the hypothesis that dietary cruciferous vegetables intake was associated with a lower risk of bladder cancer. Further large prospective cohort studies are warranted to confirm our preliminary findings.

## Introduction

Bladder cancer is a common disease, which ranks ninth in cancer incidence and is the 13th leading cause of cancer death among men and women worldwide (1). An estimated 573,278 new cases and 212,536 deaths from bladder cancer occurred in 2020 (2). Bladder cancer is classified as muscle-invasive bladder cancer (MIBC) and non-muscle-invasive bladder cancer (NMIBC) based on depth of tumor invasion. 75% of bladder cancers are non-muscle invasive (Tis, Ta, T1) (3, 4). Smoking is the most established risk factor for bladder cancer, among other risk factors including occupational exposure to arylamines and schistosomal infection (5). Emerging evidence indicates a significant influence of dietary factors [e.g., dairy product (6) and processed meat (7)] and dietary patterns [e.g., Western diet and Mediterranean diet (8)] on the risk of bladder cancer.

Cruciferous vegetables (e.g., broccoli, cauliflower, and cabbage) intake has been associated with multiple health outcomes (9). Epidemiologic studies investigating the association between cruciferous vegetables intake and bladder cancer risk, however, have yielded inconsistent results. Several case-control studies (10, 11), as well as a cohort study (12), reported a significant inverse association between cruciferous vegetables intake and bladder cancer risk. Nevertheless, many other studies found no association (13–15), including an international pooled study (15). Therefore, the aim of the present meta-analysis is to summarize the evidence on the association between cruciferous vegetables intake and bladder cancer risk based on all available cohort studies.

## Materials and methods

### Publication search

A comprehensive literature search of relevant articles was performed in the PubMed and EMBASE databases from their inception through March 2022 with the following search algorithm: (diet or nutrition or vegetable or cruciferous or broccoli or cauliflower or cabbage) and (bladder neoplasm or bladder cancer) and (cohort or prospective or nested case-control). The cited references from retrieved articles and reviews were also checked for additional relevant studies. No language restriction was applied. This systematic review and meta-analysis was planned, performed, and reported according to the standards of quality for reporting meta-analyses (16, 17).

### Study selection

Studies included in this meta-analysis met all of the following criteria: (*i*) the exposure of interest was the cruciferous vegetables intake; (*ii*) the outcome of interest was bladder cancer incidence; (*iii*) the study design was prospective or cohort; and (*iv*) the risk estimates with their corresponding 95% confidence intervals (CIs) were reported or data were provided to calculate them. We excluded reviews/meta-analyses, editorials, correspondences, case reports, and non-human studies. Studies of other exposures and diseases were also removed. If multiple publications based on overlapping population were retrieved, the most informative one was included.

### Data extraction

Two authors (PY and YL) independently extracted the data using a predefined extraction form. The following information was extracted from each study: the first author’s name, year of publication, study region, study name, study population or source, sample size (number of cases and participants), participants’ age and sex, follow-up time, method of diet assessment, method of outcome assessment, and adjusted confounders in the data analysis.

### Quality assessment

The same two authors (PY and YL) independently completed the quality assessment using the Newcastle-Ottawa Scale (NOS).^1^ NOS is an eight-item instrument and awards a maximum of nine points to each study. A higher score indicates better methodological quality. Any disagreements were resolved by consensus and discussion.

### Statistical methods

The summary RRs and 95% CIs were estimated using a DerSimonian and Laird random effects model (18). Summary risk estimates were estimated by comparing the two extreme categories of the cruciferous vegetables intake related to bladder cancer risk.

Dose-response meta-analysis was performed using the method proposed by Greenland (19) and Orsini (20). Briefly, this method required that studies reported categorical risk estimates for at least three quantitative levels of cruciferous vegetables intake and the number of cases and person-years in each exposure category. The median/mean value or the midpoint of each category was regarded as the corresponding exposure dose. For upper, open-ended exposure categories, we assumed the width of the interval to be the same as the closest neighboring category. The lowest category of exposure was treated as the reference group. In addition, we tested a potential non-linear dose-response relationship between the cruciferous vegetables intake and bladder cancer risk by modeling the cruciferous vegetables intake using restricted cubic splines with three knots at the 10th, 50th, and 90th percentiles of the distribution (21). A *P*-value for non-linearity was calculated by testing the null hypothesis that the coefficient of the second spline was equal to 0.

The heterogeneity across studies was assessed by the *Q* statistic and the *I*^2^ score (22). The *Q* statistic was used to determine the presence of heterogeneity with a significance level set at *P* ≤ 0.10. The value of *I*^2^ was used to calculate the proportion of variation (*I*^2^ < 25% low heterogeneity; *I*^2^ = 25–50% moderate heterogeneity; *I*^2^ > 50% high heterogeneity). The subgroup analyses were performed based on study region, study year, gender, number of cases, and number of participants. A sensitivity analysis was performed by repeating the meta-analysis after exclusion of each included study in turn. Potential publication bias was assessed by Begg’s test (rank correlation method) (23) and Egger’s test (linear regression method) (24). All of the statistical analyses were performed using STATA 11.0 (StataCorp, College Station, TX). A 2-sided *P*-value < 0.05 was considered significant unless stated otherwise.

## Results

### Literature search and study characteristics

**FIGURE 1:**
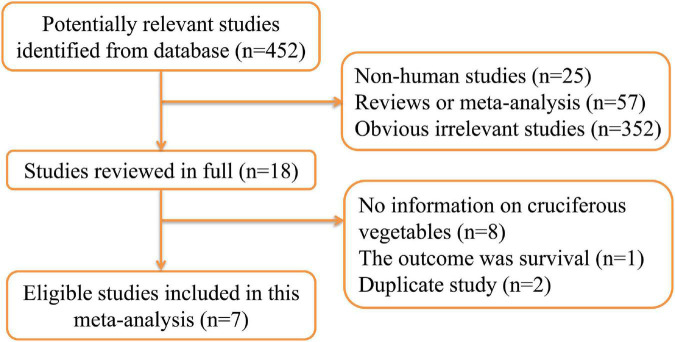
Flow diagram of literature search and study selection.

The detailed process of the literature search and selection has been presented in a flow diagram (Figure 1). A total of seven prospective studies (12–15, 25–27) met the inclusion criteria for this meta-analysis evaluating the association between cruciferous vegetables intake and bladder cancer risk. Park et al.’ study (13) reported the results separately by gender and thus was regarded as two independent cohorts. These cohorts were from the following regions: America (*n* = 5), Europe (*n* = 2), and International (*n* = 1). A total of 1,503,016 participants with 13,669 cases were included in this study. These studies were published from 1999 to 2021. Information on cruciferous vegetables consumption was obtained by self-reports or interviews with food-frequency questionnaires (FFQs). The outcome was collected from cancer registry, health insurance records or medical records. The study quality as assessed by the NOS was generally high. Only one study Larsson et al. (25) study had a score of 7, with all the other studies having a score of 8 (Supplementary Table 1). The detailed information of the studies at baseline are shown in Table 1.

**TABLE 1:** Main characteristic of included studies.

References	Name	Region	Cohort	Case	Gender	Age (y)	Follow-up (y)	Expose	Outcome
Nguyen et al. (14)	NIH-AARP diet and health study	United States	515,628	8,567	Male and female	50–71	15	Self-administered FFQ	Cancer registry
Yu et al. (15)	BLEND	International	555,685	3,203	Male and female	NA	11 (Median)	Self-administered or trained interviewer administered FFQ	Cancer registries, health insurance records, or medical records
Park et al. (13)	Multiethnic cohort study	United States	83,694	429	Male	60.2 (8.9)	12.5 (Mean)	FFQ	Cancer registry
Park et al. (13)	Multiethnic cohort study	United States	102,191	152	Female	59.7 (8.9)	12.5 (Mean)	FFQ	Cancer registry
Larsson et al. (25)	Swedish mammography cohort	Sweden	82,002	485	Male and female	NA	9.4 (Mean)	Self-administered FFQ	Cancer registry
Holick et al. (26)	Nurses’ health study	United States	88,796	237	Female	30–55	20	FFQ	Medical records
Michaud et al. (27)	ATBC cohort	Finland	27,111	344	Male	50–69	11 (Median)	FFQ	Cancer Registry
Michaud et al. (12)	HPFS	United States	47,909	252	Male	40–75	NA	Self-administered FFQ	Medical records

Y, year; FFQ, food-frequency questionnaire; NA, not available; BLEND, Bladder Cancer Epidemiology and Nutritional Determinants; HPFS, Health Professionals Follow-up Study; ATBC, Alpha-Tocopherol, Beta-Carotene Cancer Prevention.

### High vs. low cruciferous vegetables intake

**FIGURE 2:**
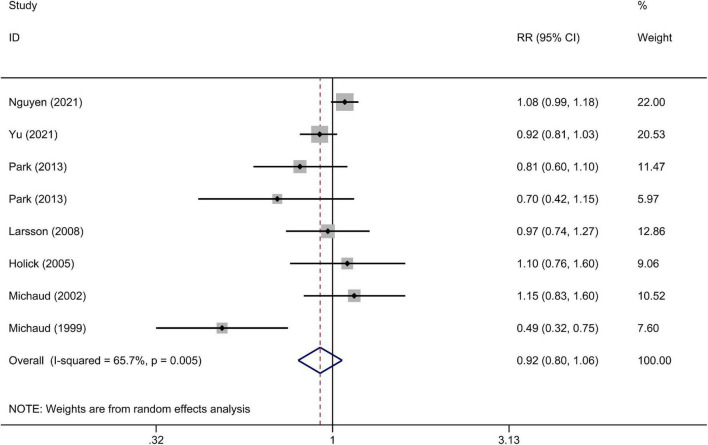
A forest plot of pooled relative risks (RRs) for cruciferous vegetables intake and bladder cancer risk (the highest category compared with the lowest category).

The multivariable-adjusted RRs of the highest vs. the lowest categories of the cruciferous vegetables intake in each study and for the combination of all of the studies are shown in Figure 2. The highest cruciferous vegetables intake was not significantly associated with a lower risk of bladder cancer, compared with the lowest category (RR = 0.92, 95% CI 0.80–1.06).

### Dose-response meta-analysis

**FIGURE 3:**
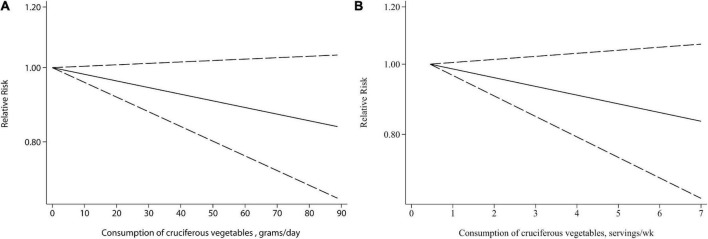
Relative risks (RRs) and the corresponding 95% confidence intervals (CIs) for the dose-response relationship between cruciferous vegetables intake and bladder cancer risk. The solid line and dash line represent the estimated RRs and their 95% CIs. **(A)** Grams per day; **(B)** servings/week.

Two units, i.e., grams/day and servings/week, were used for cruciferous vegetables intake in the included studies and thus the dose-response analysis was performed separately. There was no evidence of a non-linear relationship between cruciferous vegetables intake and bladder cancer risk (*P* = 0.169 and *P* = 0.708 for non-linearity, respectively). Linear dose-response meta-analysis indicated that neither 10 g/day or 1 servings/week increment of cruciferous vegetables intake was significantly associated with a reduced risk of bladder cancer (Figure 3, *P* = 0.106 and *P* = 0.147, respectively).

### Evaluation of heterogeneity and subgroup analysis

There was some statistically significant heterogeneity among the studies, either assessed by *Q* statistic (*P* = 0.005) or *I*^2^ index (*I*^2^ = 65.7%). In the stratified analyses, no significant association was observed in any pre-specified subgroups (Table 2).

**TABLE 2:** Subgroup analyses for the relationship between consumption of cruciferous vegetables and the risk of bladder cancer.

Factors stratified	No. of cohorts	RR (95% CI)	Q	*P*	*I*^2^, %
All cohorts	8	0.92 (0.80–1.06)	20.40	0.005	65.7
**Region**					
Europe	2	1.04 (0.84–1.28)	0.62	0.432	0.0
United States	5	0.83 (0.63–1.11)	17.61	0.001	77.3
**Publication year**					
≥2010	4	0.94 (0.81–1.10)	8.67	0.034	65.4
<2010	4	0.90 (0.65–1.25)	11.17	0.011	73.2
**Gender**					
Male	4	0.84 (0.64–1.09)	10.47	0.015	71.3
Female	3	0.91 (0.76–1.08)	2.08	0.353	3.8
**Participants, *n***					
≥100,000	3	0.97 (0.82–1.14)	6.66	0.036	70.0
<100,000	5	0.88 (0.68–1.14)	11.84	0.019	66.2
**Cases, *n***					
≥1,000	2	1.00 (0.86–1.18)	4.53	0.033	77.9
<1,000	6	0.86 (0.68–1.08)	12.76	0.026	60.8

No., number; RR, relative risk; CI, confidence interval.

### Sensitivity analysis and publication bias

**FIGURE 4:**
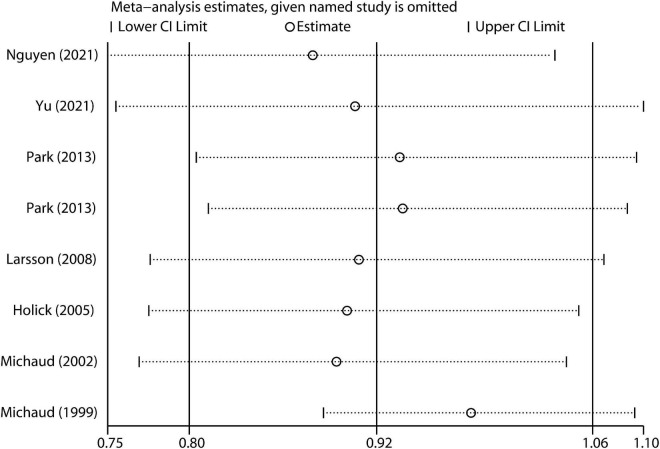
Sensitivity analysis of included studies.

**FIGURE 5:**
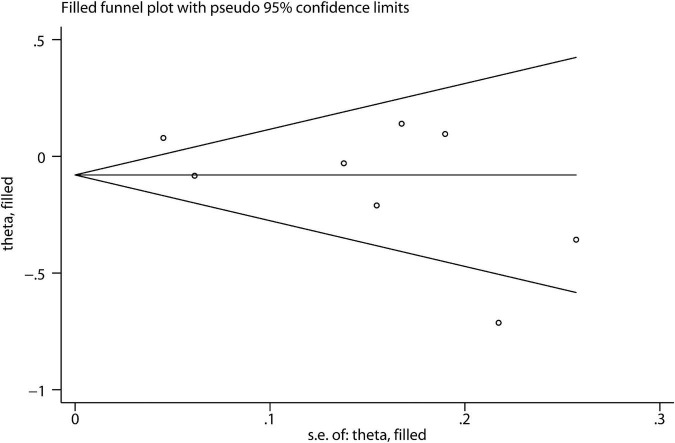
Publication bias as assessed by Begg’s test.

In the sensitivity analysis, the impact of each study on the pooled RR was evaluated by repeating the meta-analysis after omitting one study at a time. As a result, exclusion of any single study did not substantially alter the pooled RR (Figure 4). There was no evidence of significant publication bias with Begg’s test (Figure 5, *P* = 0.386) or with Egger’s test (*P* = 0.253).

## Discussion

This meta-analysis of prospective studies, including approximately 1,500,000 participants, showed that cruciferous vegetables intake was not significantly associated with the risk of bladder cancer, with consistent findings from the dose-response analysis and subgroup analysis.

A previous meta-analysis, published in 2013 by Liu et al. (28), exploring the association between cruciferous vegetables intake and bladder cancer risk was based on five cohort and five case-control studies. In the analysis of highest vs. lowest levels, cruciferous vegetables intake was significantly associated with a lower bladder cancer risk (RR 0.80, 95% CI 0.69–0.92). However, in a subgroup analysis of cohort studies the significant association did not hold, with a RR of 0.86 (95% CI, 0.61–1.11) which was pretty similar with our findings. Besides, the earlier meta-analysis did not examine the dose-response relationship and the sample size was relatively limited. By contrast, our meta-analysis included the recent studies, which further increased the sample size and improved the statistical power. In addition, only prospective studies were included in our study, which avoided the select or recall bias from case-control studies.

Cruciferous vegetable intake has been associated with multiple health outcomes. Recently, Li et al. (9) performed an umbrella review of 41 systematic reviews and meta-analyses of 303 observational studies. It revealed that cruciferous vegetable intake might have beneficial effects on several outcomes, including gastric cancer, lung cancer, endometrial cancer, and all-cause mortality. Similarly, another umbrella review of meta-analyses and systematic reviews reported that consumption of cruciferous vegetable was associated with a reduced risk of death from any cause, cancers, and depression (29). The inverse association between cruciferous vegetable intake and mortality was also supported by a large prospective cohort study with a median follow-up of 16.9 years. HR (95% CIs) for all-cause mortality in the highest compared to the lowest quintile was 0.86 (0.80–0.93) for men (*P* = 0.0002 for trend) and 0.89 (0.81–0.98) for women (*P* = 0.03 for trend) (30).

Previously several mechanisms have been proposed to explain the potential relationship between dietary cruciferous vegetables intake and bladder cancer risk. Sulforaphane, an isothiocyanate, presents naturally in cruciferous vegetables and acts as a chemopreventive agent (31). Sulforaphane plays an important role in ROS (reactive oxygen species) and ROS-related pathways, which are associated with the initiation and progression of bladder cancer (32). He et al. (33) found that the inhibitory effect of Sulforaphane on bladder cancer cells also depends on GSH (glutathione, r-glutamyl cysteingl + glycine) depletion induced by Nrf2 translocation. Xia et al. (34) reported that Sulforaphane suppressed non-muscle-invasive bladder cancer cells proliferation through inhibition of HIF-1α-mediated glycolysis in hypoxia. Benzyl isothiocyanate, another isothiocyanate presented in cruciferous vegetables, also has been reported to prevent bladder cancer progression by suppressing IGF1R, FGFR3, and mTOR (35). Abbaoui et al. (36) proposed that cruciferous vegetable isothiocyanates, including sulforaphane (SFN) and erucin (ECN), may suppress bladder carcinogenesis *via* epigenetic modulation of gene expression associated with histone H1 phosphorylation. Although various isothiocyanates from cruciferous vegetables have been proved to exert a promising anticancer effect from a substantial amount of scientific research, our study, as well as many previous epidemiological studies, did not support that cruciferous vegetable intake was associated with the bladder cancer risk.

Our meta-analysis has several strengths. First, the present study had large sample size and statistical power and only prospective studies were included. Second, the methodological quality of the included studies was generally high as assessed by NOS. Third, both categorical analysis and dose-response analysis were performed with consistent results, indicating that the findings were robust and sound. However, several limitations should also be noted. First, the number of eligible studies was still limited and no Asian studies were available. Second, although no significant publication bias was detected as assessed either by Begg’s test or Egger’s test, some publication bias may still exist as small studies with null results were less likely to be published. Third, the methods of cruciferous vegetables assessment and the cut-off points used differed across the included studies, which might distort the pooled results. Finally, a significant heterogeneity was observed among the included studies, which may weaken the robustness of the conclusion.

In summary, the results of the current study did not support the hypothesis that dietary cruciferous vegetables intake was associated with a lower risk of bladder cancer. Further large prospective cohort studies are still warranted to confirm our preliminary findings.

## Data availability statement

The raw data supporting the conclusions of this article will be made available by the authors, without undue reservation.

## Author contributions

PY and YL contributed to the conception or design of the work and drafted the manuscript. PY, LY, and YL contributed to the acquisition, analysis, and interpretation of data for the work. YL critically revised the manuscript. All authors gave final approval and agreed to be accountable for all aspects of work ensuring integrity and accuracy.

## Footnotes

1
http://www.ohri.ca/programs/clinical_epidemiology/oxford.asp
