# Supplementary material for: Dietary consumption of cruciferous vegetables and bladder cancer risk: A systematic review and meta-analysis
Source: Front Nutr. 2022 Aug 18;9:944451. doi: 10.3389/fnut.2022.944451 (PMC9434151; doi:10.3389/fnut.2022.944451)
Supplement: Supplementary file 1 [file Table_1.DOCX]

**Table S1. Methodological quality of the included studies**

| Study^a^ | Representativeness of the exposed  cohort | Selection of the  unexposed  cohort | Ascertainment  of exposure | Outcome of  interest not  present at  start of study | Comparability^b^ | Outcome  assessment | Follow-up long enough for outcomes to occur^c^ | Adequacy  of follow-up  of cohorts^d^ | Total  quality  scores |
| --- | --- | --- | --- | --- | --- | --- | --- | --- | --- |
| Nguyen et al., 2021 | ☆ | ☆ | - | ☆ | ☆☆ | ☆ | ☆ | ☆ | 8 |
| Yu et al., 2021 | ☆ | ☆ | - | ☆ | ☆☆ | ☆ | ☆ | ☆ | 8 |
| Park et al., 2013 | ☆ | ☆ | - | ☆ | ☆☆ | ☆ | ☆ | ☆ | 8 |
| Larsson et al., 2008 | ☆ | ☆ | - | ☆ | ☆☆ | ☆ | - | ☆ | 7 |
| Holick et al., 2005 | ☆ | ☆ | - | ☆ | ☆☆ | ☆ | ☆ | ☆ | 8 |
| Michaud et al., 2002 | ☆ | ☆ | - | ☆ | ☆☆ | ☆ | ☆ | ☆ | 8 |
| Michaud et al., 1999 | ☆ | ☆ | - | ☆ | ☆☆ | ☆ | ☆ | ☆ | 8 |

^a^ A study can be awarded a maximum of one star for each item except the item ‘Comparability’.

^b^ A maximum of two stars can be awarded for this item. Studies adjusted for age, sex, smoking, and total energy intake received one star while studies additionally controlling for other important confounders received an additional star.

^c^ A cohort study with a follow-up time more than 10 years was assigned one star.

^d^ A cohort study with a follow-up rate more than 75% was assigned one star.
